# A Devastating Neurological Disorder: Anti-Dipeptidyl-Peptidase-Like Protein 6 (DPPX) Encephalitis Causing Rapidly Progressive Dementia

**DOI:** 10.7759/cureus.51123

**Published:** 2023-12-26

**Authors:** Aimalohi Esechie, Neeharika Thottempudi, Chilvana Patel, Elena Shanina, Xiangping Li

**Affiliations:** 1 Neurology, University of Texas Medical Branch, Galveston, USA

**Keywords:** prion disease, startle myoclonus, autoimmune encephalitis, rapidly progressive dementia, dppx antibody

## Abstract

Rapidly progressive dementia (RPD) is caused by a heterogeneous group of neurological disorders, and the prototype is Creutzfeldt-Jakob disease (CJD). However, treatable causes including autoimmune encephalitis are often underrecognized and undertreated.

A 72-year-old female patient was admitted with a 10-month history of rapidly progressive cognitive decline, visual hallucinations, paranoid behavior, diarrhea, and an 18-kg unintentional weight loss. On the physical exam, she was only oriented to the person and demonstrated an exaggerated startle response with diffuse rigidity. The initial clinical suspicion included CJD versus autoimmune encephalitis.

Comprehensive laboratory testing, thyroid peroxidase, thyroglobulin antibodies, and autoimmune encephalitis panel were negative. The EEG showed mild to moderate diffuse slowing without any epileptiform abnormalities. An MRI brain revealed mild hippocampal atrophy. CSF testing revealed mild lymphocytic pleocytosis; RT-QuIC analysis and 14-3-3 protein were negative. There was no clinical improvement after treatment with IV steroids and IVIG. Repeated autoimmune encephalitis panel testing performed on a research basis was positive for dipeptidyl-peptidase-like protein 6 (DPPX) antibodies in serum and CSF. Unfortunately, our patient passed away before additional treatment could be attempted.

Anti-DPPX encephalitis is a rare autoimmune disorder and an unrecognized cause of RPD. Early diagnosis and rapid escalation of treatment are imperative to avoid devastating neurological consequences.

## Introduction

Dipeptidyl-peptidase-like protein 6 (DPPX), a transmembrane regulatory subunit on the Kv4.2 voltage-gated potassium channels, is widely expressed throughout the cerebellum, hippocampal pyramidal neurons, striatum, and enteric neurons [1-3]. Functionally, DPPX has been thought to mediate both membrane expression of Kv4.2 channels and signal conductance, thereby modulating dendritic excitability [1]. The presence of anti-DPPX antibodies results in a rare autoimmune encephalitis presentation that is heralded by diarrhea and weight loss. After several months or more, this is then followed by subacute neuropsychiatric symptoms characterized by cognitive dysfunction, agitation, hallucinations, and an exaggerated startle response. In many cases, resting tremors, rigidity, myoclonus, seizures, and REM sleep behavior disorders have been observed [3,4].

The clinical course of typical Creutzfeldt-Jakob disease (CJD) cases is characterized initially by nonspecific symptoms such as fatigue, unsteadiness, psychiatric symptoms (anxiety, depression), visual disorders, and memory disturbance. This is followed by a stage of rapid progressive cognitive impairment and myoclonus, eventually leading to an akinetic mutism state [5]. There has been increased awareness of CJD, which has led to the misdiagnosis of cases of rapidly progressive dementia (RPD). In a case series from prion disease referral centers [6], 8.8-47.1% of cases with suspected CJD had other identified causes of RPD, including immune-mediated, vascular, granulomatous, and neurodegenerative diseases. Although atypical presentations of neurodegenerative diseases accounted for most of the mimickers, there are autoimmune/inflammatory and other potentially treatable mimickers of CJD. Autoimmune encephalitis is an important and potentially treatable mimicker of prion disease with cases of NMDA receptor (NMDAR) encephalitis, α-amino-3-hydroxy-5-methyl-4-isoxazolepropionic acid receptor (AMPAR) encephalitis, leucine-rich glioma-inactivated 1 (LGI1) antibody-associated encephalitis, GABA-A receptor (GABAAR), GABA-B receptor (GABABR), and type 1 anti-neuronal nuclear antibody (ANNA-1)-associated encephalitis described in the literature. DPPX encephalitis has not been reported yet as a mimicker. Here, we present a case of delayed hospital presentation of a patient with DPPX encephalitis with RPD. Additionally, we discuss the diagnostic challenges of DPPX encephalitis and the prognostic implications for patients with refractory disease.

This article was previously presented as a meeting abstract at the American Academy of Neurology's (AAN) 74th Annual Meeting in April 2022.

## Case presentation

A 72-year-old Caucasian woman with a history of hypertension, recurrent urinary tract infections, age-related macular degeneration, ovarian teratoma (resected in the late 1990s), prior alcohol abuse, and osteoporosis presented for evaluation of altered mentation and rapid functional decline for over eight months. Her initial symptoms began with severe anxiety, a depressed mood, diarrhea, insomnia, and anorexia. Despite quitting alcohol, she developed worsening anxiety, mild memory impairment, an 18-kg weight loss, diaphoresis, palpitations, and labile blood pressure over the latter six months, resulting in hospital admission to an outside facility. During that hospitalization, extensive gastrointestinal and surgical endocrinology evaluations, including urine catecholamines and DOTATATE PET-CT, were unrevealing for possible neuroendocrine neoplasms. A mini-mental state exam at an outside hospital revealed a score of 21/30. Her symptoms progressed with visual hallucinations, paranoid behavior, and an exaggerated startle response. Three weeks before admission to our hospital, she became bedridden and was unable to make any meaningful conversation. Clinical examination demonstrated prominent hyperekplexia to tactile and verbal stimulation, orientation only to self, and poor attention. Diffuse muscle rigidity in her upper extremities, tremulousness, and hyperreflexia were also noted on the physical exam.

**Figure 1:**
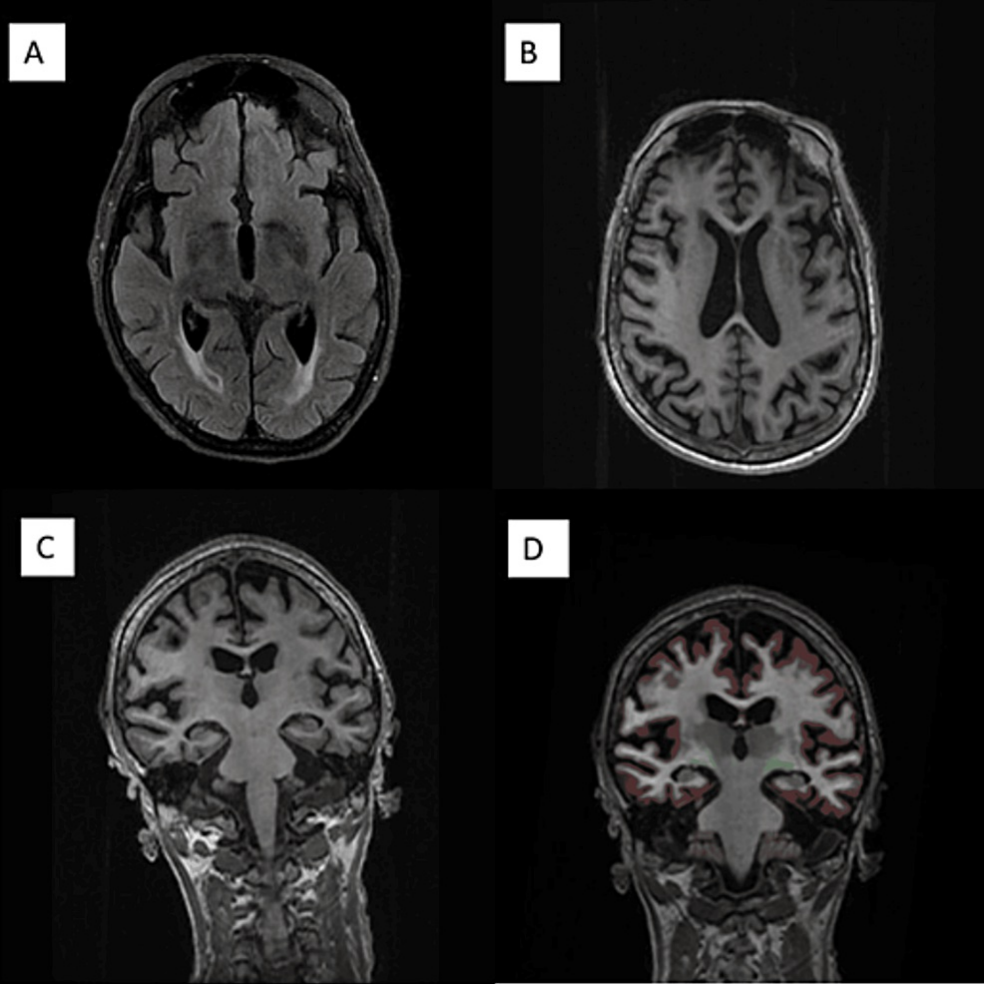
MRI brain with and without contrast at nine-month post-symptom onset (A) Axial T2 FLAIR images demonstrating nonspecific peri ventricular ischemic changes. (B) Axial T1 post-contrast images showing no evidence of enhancement. (C and D) Coronal T1 and NeuroQuant MRI demonstrating statistically significant hippocampal atrophy

Laboratory testing revealed serum sodium at 132 mmol/L; vitamin B1 and B12 and TSH were within normal limits. Syphilis and HIV testing, as well as the serum autoimmune encephalitis panel (Table 1; ARUP Laboratories), thyroid peroxidase, and thyroglobulin antibodies, were negative. Brain MRI with and without contrast revealed peri-ventricular ischemic changes and hippocampal atrophy (Figure 1). NeuroQuant volumetric processing demonstrated hippocampal atrophy (5.24 cc, 40 normative percentile) with compensatory lateral ventricle enlargement. An EEG revealed mild, diffuse slowing. CT imaging of the chest, abdomen, and pelvis was negative for underlying malignancy. The CSF examination demonstrated seven WBC/μL (88% lymphocytes) and mildly elevated protein (67 mg/dL). The oligoclonal band profile (ARUP Laboratories) is summarized in Table 1. CSF albumin was 23 mg/dL (normal 0-35 mg/dL), four oligoclonal bands were reported (normal 0-1), and the IgG index was elevated (11.2; normal 0-9). CSF glucose, microbiology, and cytology were unremarkable. At the time of serology testing for autoimmune encephalitis in serum and CSF using an extended autoimmune encephalitis panel (Table 2; ARUP Laboratories), the anti-DPPX antibody was not commercially available. ARUP Laboratories reported that our patient’s serum and CSF samples were positive when used to validate DPPX antibodies for a commercial cell-based assay with indirect immunofluorescence. Staining was observed when our patient’s specimens were incubated on brain tissue sections which was suggestive of DPPX antibodies in both specimen types. This testing was performed on a research basis. Serum titers using the extended autoimmune encephalitis panel were undetectable (anti-glutamic acid decarboxylase, anti-NMDAR, anti-AMPAR, anti-aquaporin-4 receptor, anti-LGI1, anti-GABAAR, anti-GABABR, anti-voltage-gated potassium channel, anti-CASPR2, anti-mGluR1, and anti-IgLON5). CSF T-tau protein was 1105 pg/mL (normal 0-1149 pg/mL). Real-time quaking-induced conversion (RT-QuIC) and 14-3-3 protein were negative (National Prion Disease Pathology Surveillance Center (NPDPSC)). Evaluation for progressive encephalomyelitis with rigidity and myoclonus showed no electrophysiological evidence (Figure 3) and a negative serum anti-glycine receptor (anti-GlyR) antibody.

**Table 1:** Serum and CSF qualitative isoelectric focusing CSF: cerebrospinal fluid, IgG: immunoglobulin G

ARUP 0081135	Components	Result	Reference Interval
	Oligoclonal bands, CSF	Positive	Negative
	Oligoclonal bands number, CSF	4 bands	0-1 bands
ARUP 0050676	Components	Result	Reference Interval
	IgG	756	768-1632 mg/dL (age-specific)
	IgG CSF	5.5	0.0-6.0 mg/dL
	Albumin, CSF	23	0-35 mg/dL
	Albumin, serum	2046	3500-5200 mg/dL
	Albumin index	11.2	0.0-9.0 ratio
	CSF IgG synthesis rate	6.1	≤8.0 mg/day
	IgG index	0.65	0.28-0.66 ratio
	CSF IgG/albumin ratio	0.24	0.09-0.25 ratio

**Table 2:** Autoimmune encephalitis serology testing in CSF and serum CSF: cerebrospinal fluid, IgG: immunoglobulin G, IV: intravenous

Specimen	Component	Result	Reference Interval
CSF, serum	N-methyl-D-aspartate receptor Ab	ND	<1:1, CSF, <1:10, serum
CSF	Paraneoplastic Abs (PCCA/ANNA) IgG	ND	None detected
CSF, serum	AMPA receptor Ab IgG screen	ND	<1:10
CSF, serum	GABA-B receptor Ab IgG screen	ND	<1:10, CSF, serum
CSF, serum	CASPR2 Ab IgG screen by IFA	ND	<1:10, CSF, serum
CSF, serum	LGI1 Ab IgG screen by IFA	ND	<1:10, CSF, serum
CSF, serum	CV2.1 antibody IgG screen	ND	<1:10, CSF, serum
CSF, serum	SOX1 antibody, IgG by immunoblot	N	Negative, CSF, serum
CSF, serum	Voltage-gated potassium channel Ab	N	Negative, CSF, serum
CSF, serum	Glutamic acid decarboxylase antibody	<5.0 IU/mL	0.0-5.0 IU/mL, CSF, serum
Serum	Neuronal antibody (amphiphysin)	N	Negative
Serum	Purkinje cell/neuronal nuclear IgG screen	ND	None detected
Serum	Striated muscle antibodies, IgG screen	ND	<1:40
Serum	MOG antibody IgG screen	ND	<1:10
Serum	SOX1 antibody, IgG by immunoblot	N	Negative
Serum	Acetylcholine binding antibody	ND	0.0-0.4 nmol/L
Serum	P/Q-type calcium channel antibody	ND	0.0-24.5 pmol/L
Serum	Titin antibody	0.29 IV	0.00-0.45 IV
Serum	N-type calcium channel antibody	ND	0.0-69.9 pmol/L
Serum	Ganglionic acetylcholine receptor antibody	ND	0.0-8.4 pmol/L

On hospital day 5, a five-day course of pulse IV methylprednisolone (1 g/day) was initiated and was followed by a five-day course of IV immunoglobulins (0.4 g/kg/day) on hospital day nine without significant clinical improvement. Our patient’s family elected for home hospice on hospital day 18. She died two months after her hospital discharge. The autopsy performed at the Case Western University NPDPSC with focused testing for CJD was negative.

## Discussion

DPPX antibody-associated encephalitis was first described in 2013 by Boronat et al. in four patients who presented with a constellation of symptoms that included agitation, confusion, myoclonus, tremors, and seizures [1,2,4,7-9]. Here, we report on a patient with a profound change in mental status, anxiety, diarrhea, CNS excitability, and an exaggerated startle response over the course of eight months. CSF pleocytosis and oligoclonal bands supported our clinical hypothesis of an autoimmune disorder and aggressive immune suppressive treatment was initiated. A diagnosis of anti-DPPX encephalitis was eventually confirmed with serum and CSF studies. However, she remained refractory to treatment and succumbed to disease. Boronat et al. reported that the follow-up of three out of their four reported patients indicated that the disorder is severe, requiring lengthy hospitalization of 10-15 months after symptoms onset to return home, and was maintained on long-term immunosuppressive therapy with multiple relapses when tapering therapy [1].

The clinical features, especially profound diarrhea, are typical of DPPX encephalitis and atypical for CJD. GI symptoms are typical in the early phase of the disease and may not be present at presentation. Studies have shown that diarrhea usually precedes the development of neurological symptoms by a median of four months [7]. Our patient presented to us later in the clinical course, thus highlighting the importance of careful history-taking and an awareness of the clinical features of DPPX encephalitis that would raise suspicion of this rare disease.

Neuroimaging findings in patients with autoimmune encephalitis often involve the limbic structures, but the involvement of the striatum, diencephalon, or rhombencephalon can also be seen. In a subset of patients, despite profound neuropsychiatric dysfunction, neuroimaging can be normal [10]. MRI brain findings commonly reported in DPPX antibody-associated encephalitis include non-specific T2 FLAIR white matter abnormalities or normal imaging [11]. A recent report by Xiao et al. demonstrated MRI changes of increased T2/FLAIR signals in the bilateral hippocampus, temporal lobe, and amygdala, similar to other autoimmune encephalitis, in seven patients with DPPX antibody-associated encephalitis [12]. In our patient, the MRI brain showed hippocampal atrophy with NeuroQuant volumetric analysis. We hypothesize that given that DPPX is predominantly expressed in the hippocampus and our patient presented late in her clinical course, the prolonged duration of inflammation may have contributed, in part, to the atrophic changes in the hippocampus. Studies on DPPX encephalitis are limited and therefore insufficient to support our hypothesis. Additionally, future investigation into the role of hippocampal atrophy in the spectrum of DPPX and other autoimmune encephalitis is warranted [13]. Whether the prolonged inflammation contributed to the refractory response to immunosuppressive therapy requires further investigation. The inciting events precipitating the onset of DPPX-encephalitis are unknown. We relate the presenting symptoms to the localization of the DPPX antibodies. Piepgras et al. demonstrated a somatodendritic, presynaptic staining pattern for both glutaminergic synapses and GABAergic synapses, suggesting excitatory and inhibitory modulation in hippocampal and cerebellar neuronal networks [8]. Specifically, these autoantibodies bind to unmyelinated nerve fibers in the hippocampal cornu ammonis 1 (CA1), thus leading, in part, to memory impairment [14].

In patients presenting with rapidly progressive cognitive deficits with neuropsychiatric symptoms, important differentials to consider include CJD, autoimmune encephalitis, and CNS vasculitis. CJD is a rare and diagnostically challenging prion disease that is often confirmed late in the illness. Our patient did meet the criteria for “probable CJD” on initial presentation to us based on rapidly progressive dementia and signs of CNS hyperexcitability. The absence of cortical ribboning and diffusion restriction within deep nuclei on neuroimaging and EEG findings was inconsistent with CJD in our patient. While there are many overlapping non-specific features of the MRI brain and EEG, characteristic MRI findings are seen in 71% of CJD patients and typical EEG findings in 19% [6]. A differential throughout the diagnosis of CJD was eliminated after further testing resulted in a negative RT-QuIC and NPDPSC neuropathology. Maat et al. previously reported that 7% of patients with suspected CJD had autoimmune encephalitis at autopsy. Antibodies that were detected include anti-Hu, GABA B1/B2, NMDAR, and CASPR2, while two CSF samples had antibodies against an unidentified neuronal surface antigen [5,15]. Rossi et al. reported that less than 5% of patients with sporadic CJD develop serum antibodies to neuronal antigens in low titers, and while their significance is unclear, they could possibly be related to rapid neuronal destruction [16]. Additionally, our patient had detectable CSF T-tau protein (within the normal range). Studies have shown that elevated CSF T-tau increases the probability of prion disease by a factor of 2.4 at 1150 pg/ml or greater [17]. Elevated T-tau protein has also been reported in autoimmune encephalitis, especially in patients with MRI temporal FLAIR signal changes and in patients developing hippocampal sclerosis [18].

Fewer than 10-30% of patients with malignancy have associated DPPX positivity, most commonly B-cell neoplasm, with few cases of spontaneous recovery from encephalitis prior to treatment of the malignancy [4,10,14]. A summary of clinical symptoms in 20 patients with positive DPPX serology by Tobin et al. further highlighted the insidious onset of symptoms and the protracted duration of symptoms before immune-suppressive therapy is administered [4]. Treatment of DPPX encephalitis is directed toward immune suppression; however, patients suffer relapses and, in some cases, have fatal outcomes despite early diagnosis [1,2,4,7,13,19]. While the anti-DPPX antibody in our patient’s serum and CSF was positive, the lack of commercially available testing at the time contributed to the delay in the escalation of immunosuppression.

## Conclusions

DPPX antibody-associated encephalitis is a rare autoimmune disorder and is a potentially treatable cause of rapid progressive dementia if recognized early in the course of the disease. Its clinical features, such as rapidly progressive cognitive decline, neuropsychiatric symptoms, rigidity, and exaggerated startle myoclonus, could mimic CJD and other neurodegenerative disorders. Anti-DPPX encephalitis can be refractory to first-line treatment. Early diagnosis and rapid escalation of treatment are imperative to avoid devastating neurological consequences.
